# GmBEHL1, a BES1/BZR1 family protein, negatively regulates soybean nodulation

**DOI:** 10.1038/s41598-018-25910-x

**Published:** 2018-05-16

**Authors:** Qiqi Yan, Lixiang Wang, Xia Li

**Affiliations:** State Key Laboratory of Agricultural Microbiology, College of Plant Science and Technology, Huazhong Agricultural University, Wuhan, 430070 P.R. China

## Abstract

Brassinosteroids (BRs) play an essential role in plant growth, and BRI1-EMS suppressor 1 (BES1)/brassinazole-resistant 1 (BZR1) family transcription factors integrate a variety of plant signaling pathways. Despite the fact that BRs inhibit nodulation in leguminous plants, how BRs modulate rhizobia-host interactions and nodule morphogenesis is unknown. Here, we show that GmBEHL1, a soybean homolog of *Arabidopsis* BES1/BZR1 homolog 1 (BEH1), is an interacting partner of Nodule Number Control 1, a transcriptional repressor that mediates soybean nodulation. *GmBEHL1* was highly expressed at the basal parts of emerging nodules, and its expression gradually expanded during nodule maturation. The overexpression and downregulation of *GmBEHL1* inhibited and enhanced the number of nodules, respectively, in soybean. Intriguingly, alterations in *GmBEHL1* expression repressed the expression of genes in the BR biosynthesis pathway, including homologs of *Arabidopsis Constitutive Photomorphogenesis and Dwarf* and *Dwarf 4*. We also detected an interaction between GmBEHL1 and GmBIN2, a putative BR-insensitive 2 (BIN2) homolog, in soybean. Moreover, BR treatment reduced the number, but increased the size, of soybean nodules. Our results reveal *GmBEHL1* to be a potent gene that integrates BR signaling with nodulation signaling pathways to regulate symbiotic nodulation.

## Introduction

Nitrogen is an essential element for plant growth and development. To obtain sufficient nitrogen, non-leguminous plants have evolved developmental plasticity of the root system that enables them to remodel their root architecture (i.e., lateral root formation) in response to fluctuating levels of nitrogen in the growth environment^1^. Given their high demand for nitrogen, leguminous plants have developed an additional form of root developmental plasticity (the formation of symbiotic root nodules) that allows plants to adapt to nitrogen-deficient conditions. Root nodules are formed from the cortical cells of a primary root according to a unique genetic program that allows a rhizobial infection to take place and *de novo* nitrogen-fixing organ formation to occur^2,3^. Thus, root nodules are capable of fixing atmospheric nitrogen to meet the demands of leguminous plants. Interestingly, indeterminate nodules in the roots of leguminous plants such as *Medicago truncatula* have a similar structure to lateral roots that includes functionally and developmentally different zones, including an apical meristematic zone, which allow the indeterminate nodules to continue to grow^3^. By contrast, the determinate nodules in roots of *Lotus japonicus* and *Glycine max* (soybean) are spherical, lateral organs with no apparent developmental zones^3^.

In recent decades, many studies have focused on the early stages of legume-rhizobial symbioses. Nodule development is trigged by a rhizobial infection, and both the initiation of rhizobial entry into roots and the onset of nodulation are dependent on the perception of nodulation factors (NFs) by LysM receptors (e.g., NF Perception [NFP] in *M. truncatula*, NF Receptors 1 and 5 [NFR1/5] in *L. japonicus*, and NFR1/5α in soybean), which activate a signaling cascade (the NF signaling pathway) that triggers nodule formation^4–9^. In *M. truncatula*, the key event following NFP binding is the accumulation of calcium within and around the nucleus of infected root hair cells mediated by Does Not Make Infections 1 and 2 (DMI2 and DMI1, respectively) and cyclic nucleotide-gated channels localized to the nuclear envelope^10–12^. Calcium oscillations are then decoded via a calmodulin 1 and calcium and calmodulin-dependent kinase-mediated network that is activated by the phosphorylation of a transcription factor (CYCLOPS) and its interacting partner (DMI3) in *L. japonicus* and *M. truncatula*, respectively^13,14^. CYCLOPS subsequently *trans*-activates Nodule Inception (NIN) and ERF Required for Nodulation1 (ERN1). NIN is a central regulator of nodulation, which targets early nodulation genes (*ENODs*) such as *ENOD11* in the root epidermis and *Cytokinin Receptor 1* (*CRE1*) in the cortex of *M. truncatula* roots to repress or activate their expression^15^. Several transcription factors belonging to the GRAS (e.g., Nodulation Signaling Pathway 1/2 [NSP1/2]); Nuclear factor Y (e.g., NF-YA1); Ethylene Response Factor ERF/APETALA2 (ERF/AP2); and NAM, ATAF1/2, and CUC2 domain protein families are also involved in nodulation^16–19^. Recently, it was shown that microRNAs, noncoding RNAs that are 20–22 nucleotides in length, are involved in regulating nodulation through the repression of their target genes^20,21^. In soybean, we identified miR172c as a key positive regulator of nodulation that promotes the cleavage of mRNAs encoding its target gene *Nodule Number Control 1* (*NNC1*), which directly suppresses the transcription of *ENOD40* and nodule development^22^. In addition, nodule number is controlled by the autoregulation of nodulation (AON) signaling pathway, which is initiated by the activation of *CLE-RS1* and *CLE-RS2* (*CLE-Root Signal1/2*) in rhizobia-infected roots during primordia formation; signaling is in turn perceived by specific receptors, including SUNN(Super Numerary Nodules)in *M. truncatula*, HAR1(Hypernodulation Aberrant Root Formation 1) in *L. japonicus*, and GmNARK (Nodule Autoregulation Recepter Kinase) in soybean^23–25^. Despite extensive progress, many questions concerning cell priming for nodule initiation, primordia formation, and nodule organogenesis remain unanswered.

It is well known that phytohormones are involved in nodule formation and development in legumes. Among them, auxin and cytokinin (CK) are the major hormones regulating root nodule development^26,27^; however, other phytohormones, including abscisic acid (ABA), strigolactones, gibberellic acid, ethylene, and jasmonic acid, and their interplay also participate in root nodule development^28–32^. Brassinosteroids **(**BRs) are a group of steroid hormones in plants that play crucial roles in shoot elongation, plant architecture, photomorphogenesis, and seed germination^33^. Because they are highly mobile, BRs can effectively regulate cell elongation and lateral organ development^33^. In *Arabidopsis*, many key components, including BR receptors, co-receptors, and their direct downstream components, have been identified and the core BR signaling pathway has been defined. BRASSINAZOLE-RESISTANT 1 (BZR1) and its homologs (BES1/BZR1-like genes [BEHs]) are key regulators of BR signaling that repress or activate the transcription of BR-responsive genes to mediate plant growth and development^34,35^. BZR1 and BEHs can directly repress the expression of genes involved in BR biosynthesis, thereby suppressing BR signaling through feedback regulation^36^. In legumes, the role of BRs in nodulation has been explored. In pea, the BR biosynthesis mutants *lk* (affecting 5α-reductase) and *lkb* (affecting sterol C-24 reductase), as well as the BR receptor mutant *lka*, all exhibit a reduced number of nodules^37^. Meanwhile, a genetic analysis of double mutants (*lk* and an AON-related mutant such as *nark*) revealed that BRs regulate nodule number in an AON-independent manner in pea plants^38^. In *M. truncatula*, a loss-of-function mutant of the BR receptor MtBRI1 (*mtbri1*) displayed a reduced number of nodules and a range of defects in symbiotic nitrogen fixation^39^. Interestingly, an earlier study indicated an opposing role for BRs in soybean nodulation; the exogenous application of brassinazole, an effective inhibitor of BR biosynthesis, resulted in an increase in nodule number in the soybean cultivar Enrei^40^. Despite limited data, there is no doubt that BRs mediate symbiotic nodulation in leguminous plants. Still, further molecular evidence is needed to address how BRs are involved in nodulation in different legumes.

In this study, we identified a soybean homolog of *Arabidopsis* BES1/BZR1 homolog (BEH1) as an NNC1-interacting protein and named it GmBEHL1. The knockdown and overexpression of *GmBEHL1* resulted in increased and decreased numbers of nodules, respectively, in composite transgenic roots. Similar to BEH1, GmBEHL1 is localized to both the cytoplasm and nucleus, and it can bind directly to BR-responsive Responsive Elements and interact with GmBIN2, a soybean homolog of *Arabidopsis* BR-insensitive 2 (BIN2). Furthermore, we uncovered the diverse roles of BRs in determining the number and size of the nodules in soybean plants. Our results suggest that GmBEHL1 functions as a co-repressor to negatively regulate soybean nodulation; moreover, they reveal for the first time direct crosstalk between the NF and BR signaling pathways in soybean.

## Results

### GmBEHL1 is an NNC1-interacting protein and homolog of *Arabidopsis* BES1/BZR1

**Figure 1:**
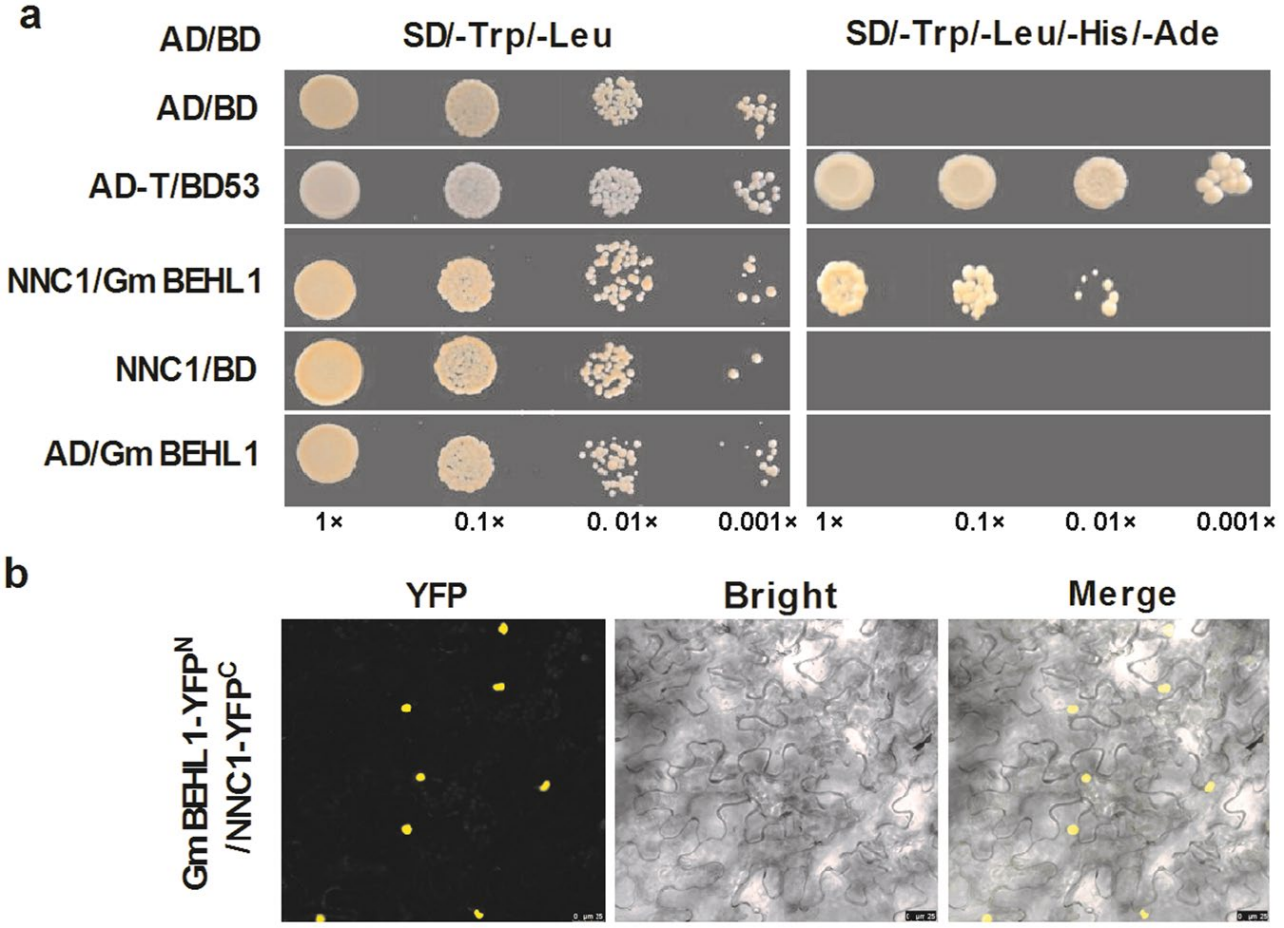
NNC1 interacts directly with GmBEHL1. (**a**) Results of Y2H assay to detect interactions between NNC1 and GmBEHL1. Yeast cells co-transformed with the constructs pGADT7/pGBKT7-GmBEHL1, pGADT7-NNC1/pGBKT7-GmBEHL1, and pGADT7-NNC1/pGBKT7 were selected and grown on selective media lacking Leu, and Trp (SD/−2); surviving cells were subsequently transferred to selective media lacking Ade, His, Leu, and Trp (SD/−4) to test for protein-protein interactions. (**b**) Results of a BiFC assay to detect the interaction of NNC1 with GmBEHL1. GmBEHL1 and NNC1 were fused to the N-terminus of YFP (nYFP) and C-terminus of YFP (cYFP), respectively. Bars = 25 μm.

Because NNC1 is a key transcriptional repressor of *ENOD40-1* that modulates soybean nodulation, we sought to identify its functional partners in order to uncover the regulatory mechanisms it mediates. To do this, we performed a yeast two-hybrid (Y2H) screen to isolate NNC1-interacting proteins. Interestingly, we found that one of the NNC1-interacting proteins (Glyma.01G178000) was an *Arabidopsis* BEH1-like protein^41^; therefore, it was named GmBEHL1. Based on this, we speculated that GmBEHL1 acts as a node in the NF and BR signaling pathways. To test this hypothesis, we first confirmed the NNC1-GmBEHL1 interaction using *in vitro* and *in vivo* protein-protein interaction assays. Our Y2H assay results showed that GmBEHL1 interacted directly with NNC1 (Fig. 1a). We next performed a bimolecular fluorescence complementation (BiFC) assay by co-expressing GmBEHL1-YFP^**N**^ and NNC1-YFP^**C**^ in *Nicotiana benthamiana* leaves. As shown in Fig. 1b, GmBEHL1 interacted strongly with NNC1 in the nucleus of each transformed cell, consistent with their transcriptional properties. These results indicate that GmBEHL1 may form a complex with NNC1 in rhizobia-inoculated soybean roots to mediate nodulation.

### *GmBEHL1* is a negative regulator of soybean nodulation

**Figure 2:**
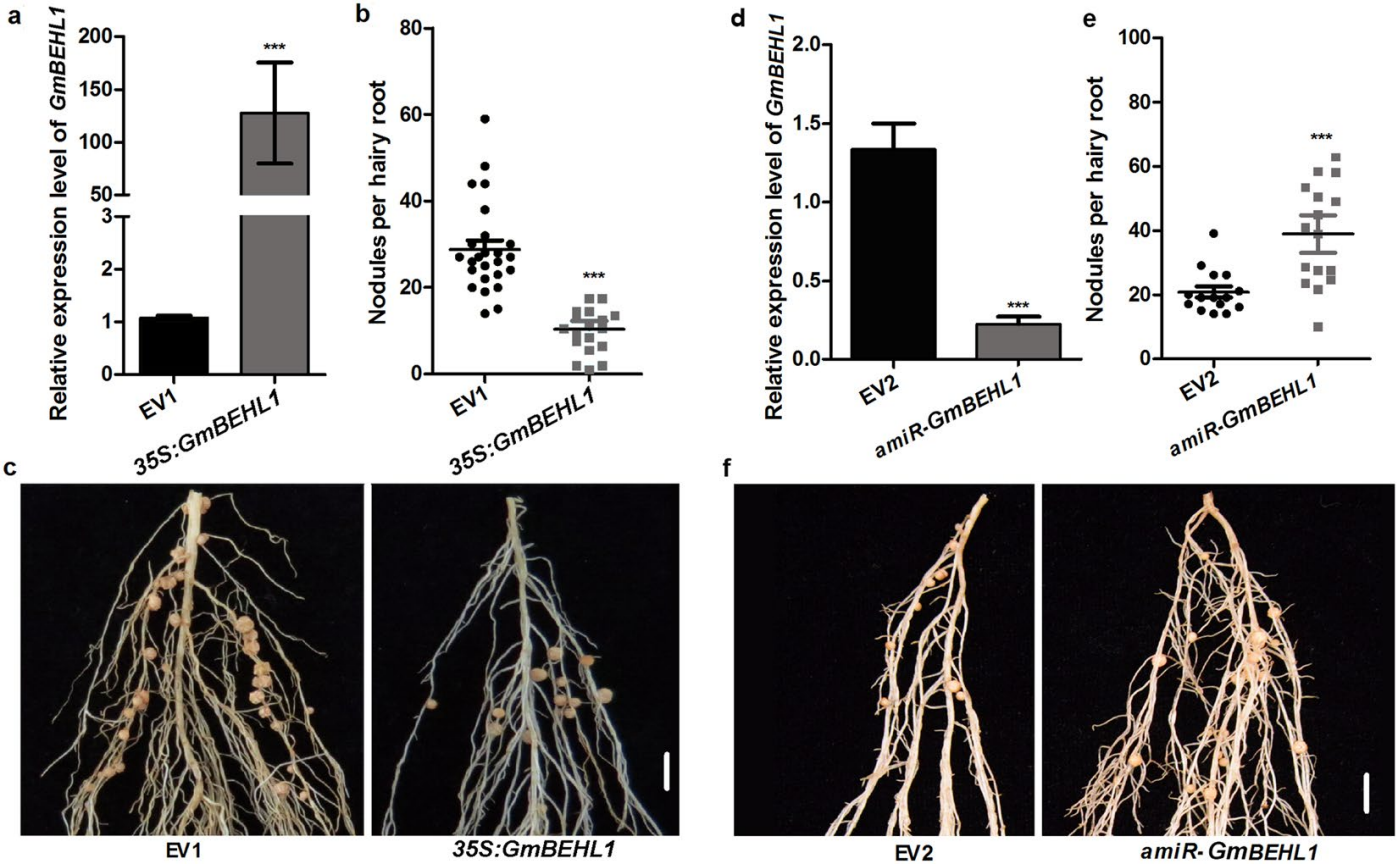
Changes in *GmBEHL1* expression affect nodulation. (**a**–**c**) *GmBEHL1* overexpression suppresses nodulation. (**a**) qRT-PCR analysis of transgenic hairy roots expressing *35S:GmBEHL1*. The relative expression levels of *GmBEHL1* in hairy roots transformed with empty vector 1 (EV1) or *35S:GmBEHL1*. The expression levels were normalized against the geometric mean of soybean *GmELF1b* for *GmBEHL1*. Student’s *t-*test was performed (***p < 0.001, n = 25). (**b**) Quantitative analysis of the nodule number per hairy root expressing EV1 and *35S:GmBEHL1* (n = 25). All values are the means ± SDs from more than three independent experiments. Asterisks represent statistically significant differences (Student’s *t*-test, ***p < 0.001). (**c**) Nodules of individual hairy roots expressing EV1 and *35S:GmBEHL1* at 28 DAI. Bar = 3 mm. (**d**–**f**) Knocking down *GmBEHL1* promotes nodulation. (**d**) Expression analysis of transgenic hairy roots expressing empty vector 2 (EV2) and *amiR-GmBEHL1*. The expression levels of *GmBEHL1* were normalized against the geometric mean of soybean *GmELF1b*. Student’s *t-*test was performed (n = 25, ***p < 0.001). (**e**) Quantitative data showing the nodules per hairy root for EV2 and the vector harboring *amiR-GmBEHL1* (n = 25). The nodule numbers are the means ± SDs from more than three independent experiments. Asterisks represent statistically significant differences (Student’s *t*-test; ***p < 0.001). (**f**) Nodules of individual hairy roots expressing EV2 and *amiR-GmBEHL1* at 28 DAI. Bar = 3 mm.

Our immediate question was whether GmBEHL1 plays a regulatory role in soybean nodulation. To address this, we performed a systemic phenotypic analysis of *GmBEHL1* overexpression or knockout/knockdown roots using the hairy root transformation system. First, we made a construct containing *CaMV35S promoter (35S):GmBEHL1* and obtained transformed roots overexpressing *GmBEHL1* (*GmBEHL1OX*) for the evaluation of nodulation; this was confirmed by checking the *Bar* gene (Fig. S1) and qRT-PCR analysis (Fig. 2a). The effects of *GmBEHL1* overexpression on the early and late stages of nodulation were evaluated at 6 and 28 days after inoculation (DAI) using *Bradyrhizobium* (*B*.) *japonicum* USDA110. The number of root hairs showing deformation was markedly decreased in *GmBEHL1OX* hairy roots at 6 DAI (Fig. S2). Transformed hairy roots overexpressing *GmBEHL1* were also used to determine nodule numbers at 28 DAI. Intriguingly, the *GmBEHL1OX* roots produced significantly fewer nodules than the control roots did (Fig. 2b and c). The average number of nodules per vector control root was 27.3, whereas the average number of nodules per *GmBEHL1OX* root was only 10.9. Thus, the total number of nodules per *GmBEHL1OX* root was reduced by approximately 60.1%.

To investigate whether endogenous *GmBEHL1* is required for nodulation, we created an *amiR-GmBEHL1* construct to knock down the *GmBEHL1* gene (Fig. 2d) and evaluated the effect of reduced *GmBEHL1* expression on nodulation (Fig. 2e and f). As shown in Fig. 2e and f, *amiR-GmBEHL1* transgenic roots with reduced expression of *GmBEHL1* produced significantly more nodules compared with vector control roots. The average number of nodules per vector control root was only 21.2, whereas the average number of nodules per *GmBEHL1-RNAi* transgenic root was 39.1. Thus, the total number of nodules per *GmBEHL1-RNAi* transgenic root was increased by approximately 84.4%. Together, these data suggest that *GmBEHL1* negatively regulates nodulation in soybean.

### Changes in *GmBEHL1* expression affect marker genes in the NF pathway

**Figure 3:**
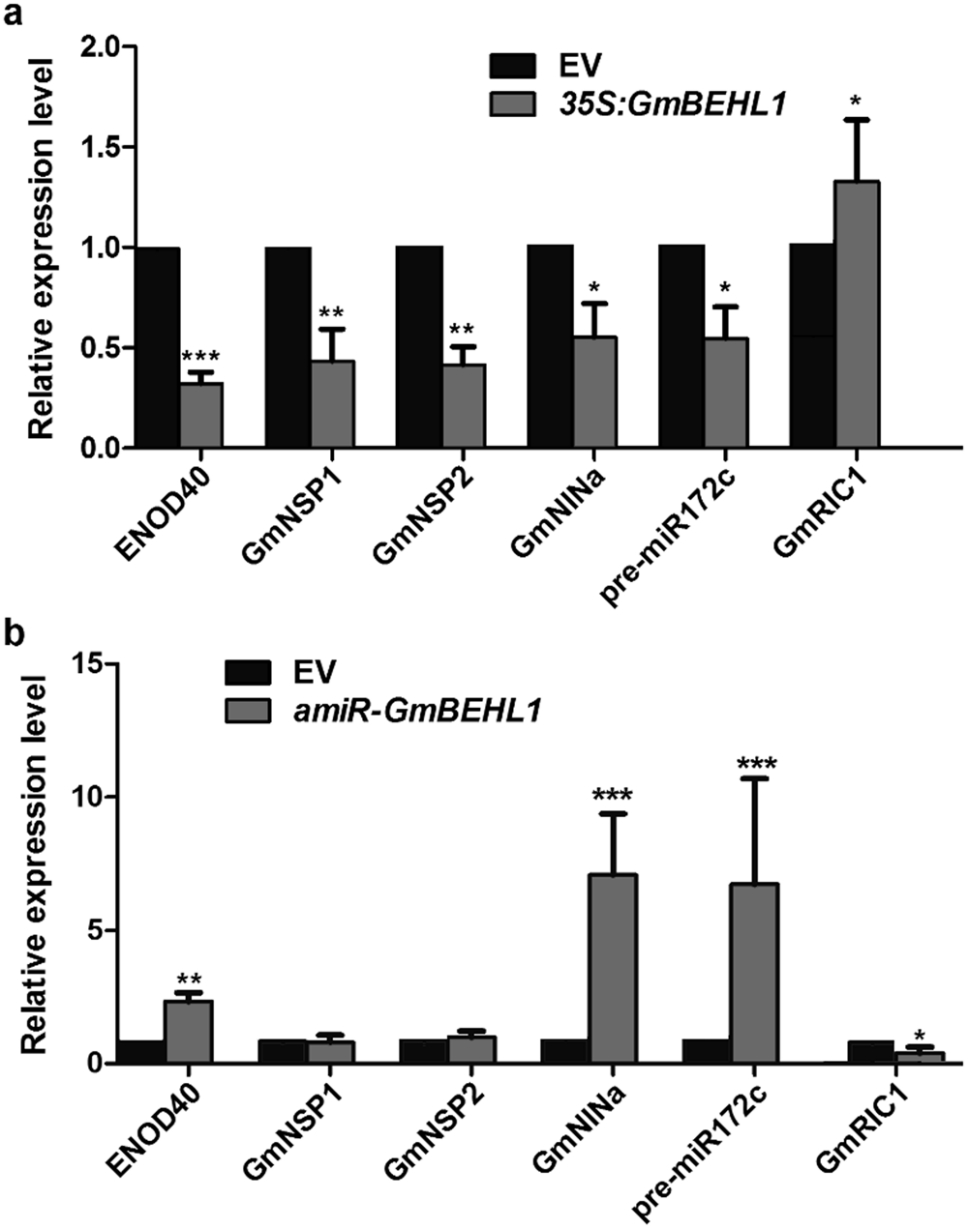
Alterations in *GmBEHL1* expression affect the transcript levels of nodulation-related genes. (**a**) qRT-PCR analysis of *ENOD40*, *GmNSP1*, *GmNSP2*, *GmNIN*, *pre-miR172c*, and *GmRIC1* in roots transformed with EV1 and *35S:GmBEHL1* at 2 DAI (n = 6). (**b**) qRT-PCR analysis of *ENOD40*, *GmNSP1*, *GmNSP2*, *GmNIN*, *pre-miR172c*, and *GmRIC1* in roots transformed with EV2 and *amiR-GmBEHL1* at 2 DAI (n = 6). We set all of the transcript profiles of the *GmENOD40*, *GmNSP1*, *GmNSP2* and *GmNINa* in 2 DAI EV hairy roots as “1”. The transcript amounts in each sample were normalized to those of *ELF1b*. The expression levels shown are the means ± SDs from three replicates. Asterisks represent statistically significant differences compared to the empty control (Student’s *t*-test; *p < 0.05, **p < 0.01, and ***p < 0.001).

Given that altering the expression of *GmBEHL1* dramatically affected the nodule number in soybean, we questioned whether *GmBEHL1* regulates soybean nodulation through the NF and AON signaling pathways. To this end, we examined the expression pattern of a number of nodulation and AON marker genes during nodulation using roots in which *GmBEHL1* was overexpressed or knocked down. The marker genes included *ENOD40-1* and functional orthologs of *LjNIN* (Glyma.04G000600 [*GmNINa*]), *LjNSP1* (Glyma.07G039400 [*GmNSP1*]), *LjNSP2* (Glyma.04G251900 [*GmNSP2*]), and miR172c in the NF signaling pathway, and *GmRIC1* in the AON signaling pathway. As shown in Fig. 3, the expression of *ENOD40-1*, *GmNINa*, *GmNSP1*, *GmNSP2*, and *pre-miR172c* in *GmBEHL1OX* roots was significantly reduced compared with that in empty vector control roots at 2 DAI, while *GmRIC1* was upregulated (Fig. 3a). In contrast, the transcript levels of *ENOD40-1*, *GmNIN*, and *pre-miR172c* in *amiR-GmBEHL1* hairy roots were markedly increased, although *NSP1*, *NSP2*, and *GmRIC1* expression was not dramatically affected (Fig. 3b). Our results suggest that *GmBEHL1* acts upstream of these symbiosis-related genes in controlling rhizobial infection and nodule development, and that *GmBEHL1* participates in nodule number regulation via the AON signaling pathway.

### *GmBEHL1* is expressed in multiple organs and is dynamically expressed during nodulation

**Figure 4:**
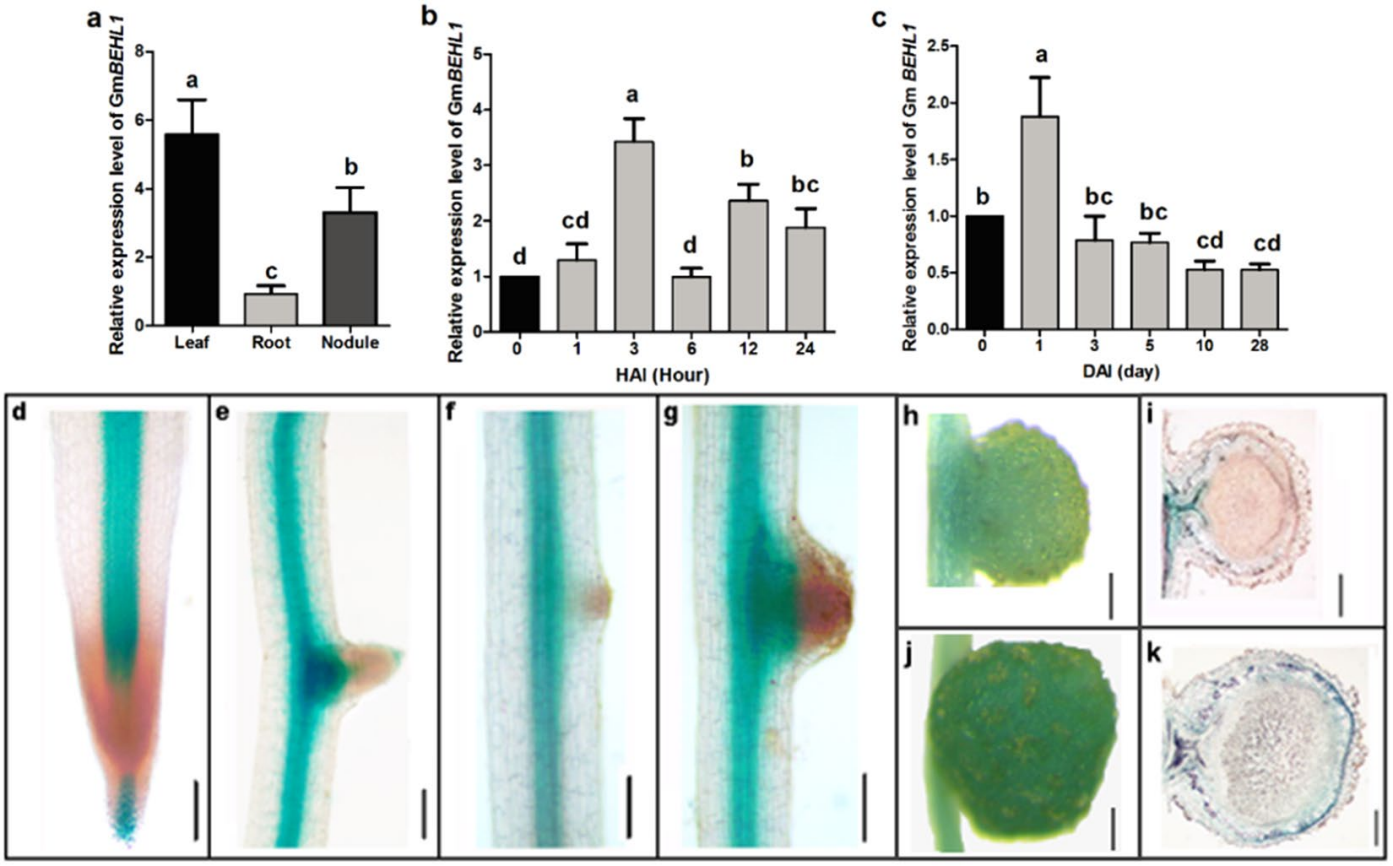
The expression pattern of *GmBEHL1*. (**a**–**c**) qRT-PCR analysis of *GmBEHL1*. (**a**) Seven-day-old seedlings were inoculated with *B. japonicum* strain USDA110; roots, leaves, and nodules harvested at 28 DAI were used for gene expression analyses (n = 15). (**b**) Inoculated roots were harvested at 0, 1, 3, 6, 12, and 24 HAI and the expression of *GmBEHL1* at the early stage of nodulation was analyzed. (**c**) The *GmBEHL1* expression pattern during nodulation was analyzed using root samples harvested at 0, 1, 3, 5, and 10 DAI (n = 15). *GmELF1b* was used as an endogenous control for gene expression. The expression levels are shown as the means ± SDs from three replicates. Means with different letters are significantly different (*p* < 0.05; Tukey’s test). (**d**–**i**) Histochemical localization of GUS activity in the transgenic hairy roots and at different stages of nodulation. (**d** and **e**) Expression pattern of *GmBEHL1pro:GUS* in primary (**d**) and lateral roots (**e**). (**f**–**i**) *GmBEHL1pro:GUS* activity at different stages of soybean nodulation. (f and g) GUS activity during nodule initiation (**f**) and primordia formation (**g**). (**h**,**i**) GUS staining of *GmBEHL1pro:GUS* in young nodule at 14 DAI (**h**) and in paraffin section of young nodule (**i**); (**j**,**k**) GUS staining of mature nodule at 28 DAI (**j**) and paraffin section of the stained mature nodule (**k**). Bars in (**d**–**k**) = 200 μm.

To validate the expression pattern of *GmBEHL1*, we performed a qRT-PCR analysis of samples collected from leaves, roots, and root nodules at 28 DAI. As expected, *GmBEHL1* was expressed in all of the tested organs, though the transcript level of the gene was highest in leaves, intermediate in nodules, and lowest in roots (Fig. 4a). Next, we measured the expression of *GmBEHL1* in roots at the early or late stages of nodulation. Within 24 h after inoculation (HAI), *GmBEHL1* was rapidly upregulated and reached its peak at 3 HAI in the roots; further, *GmBEHL1* expression was restored to its original level before another peak (Fig. 4b). Interestingly, the *GmBEHL1* expression level started to decline at 3 DAI and dropped continously, exhibiting its lowest level when the nodules reached maturity (Fig. 4c). The dynamic pattern of *GmBEHL1* expression during nodulation suggests diverse roles for the gene during nodulation in soybean.

To further validate the transcriptional actitivty of the *GmBEHL1* promoter, we made a construct harboring *GmBEHL1pro:GUS* and generated transformed hairy roots expressing *GmBEHL1pro:GUS* for a GUS assay. In uninfected hairy roots, *GmBEHL1* was expressed at high levels in the root cap and mature regions; in sharp contrast, there was no visible expression of *GmBEHL1* in the apical meristems of the primary and lateral roots (Fig. 4d and e). The expression pattern of *GmBEHL1* in hairy roots inoculated with rhizobia was not significantly different from that in uninfected roots. However, increased expression of *GmBEHL1* was observed in nodule primordia; moreover, when the nodules emerged, strong *GmBEHL1* expression was observed only in the basal parts of the emerging and developing nodules (Fig. 4f–i). Interestingly, GUS staining of young and mature nodules showed gradual expansion of *GmBEHL1* expression from the basal parts to the top of each nodule during nodule development. In addition, *GmBEHL1* was expressed throughout the tissues of fully developed nodules, with the highest level of expression observed in the vascular bundles of the nodules (Fig. 4j and k). Our results confirm that *GmBEHL1* participates in various processes during nodulation and root development.

### GmBEHL1 is a nucleocytoplasmic protein with DNA-binding activity

Since GmBEHL1 is annotated as a BEH1-related protein, we conducted a phylogenetic analysis to verify the relationship between GmBEHL1 and its homolog in *Arabidopsis*. Our results confirmed that GmBEHL1 is the closest relative of BEH1 (Fig. S3a). GmBEHL1 shares high levels of amino acid sequence identity and structural similarities with *Arabidopsis* BEH1 (Fig. S3b). Both proteins have an N-terminal BES1_N domain, which is typical of BES1/BZR1 family proteins, and other domains, including a nuclear localization signal, P domain, and PEST domain^36^.

To assess the localization of GmBEHL1 in plant cells, we produced a construct harboring the *35S:GmBEHL1-GFP* expression cassette and expressed the GmBEHL1-GFP fusion protein in *N. benthamiana* leaves. Confocal laser scanning microscopy revealed that GmBEHL1-GFP was localized in both the cytoplasm and nucleus (Fig. S3c), consistent with previous findings on the subcellular localization of *Arabidopsis* BZR1, implying that GmBEHL1 is a nucleocytoplasmic shuttling protein, like *Arabidopsis* BZR1 family proteins^42^.

**Figure 5:**
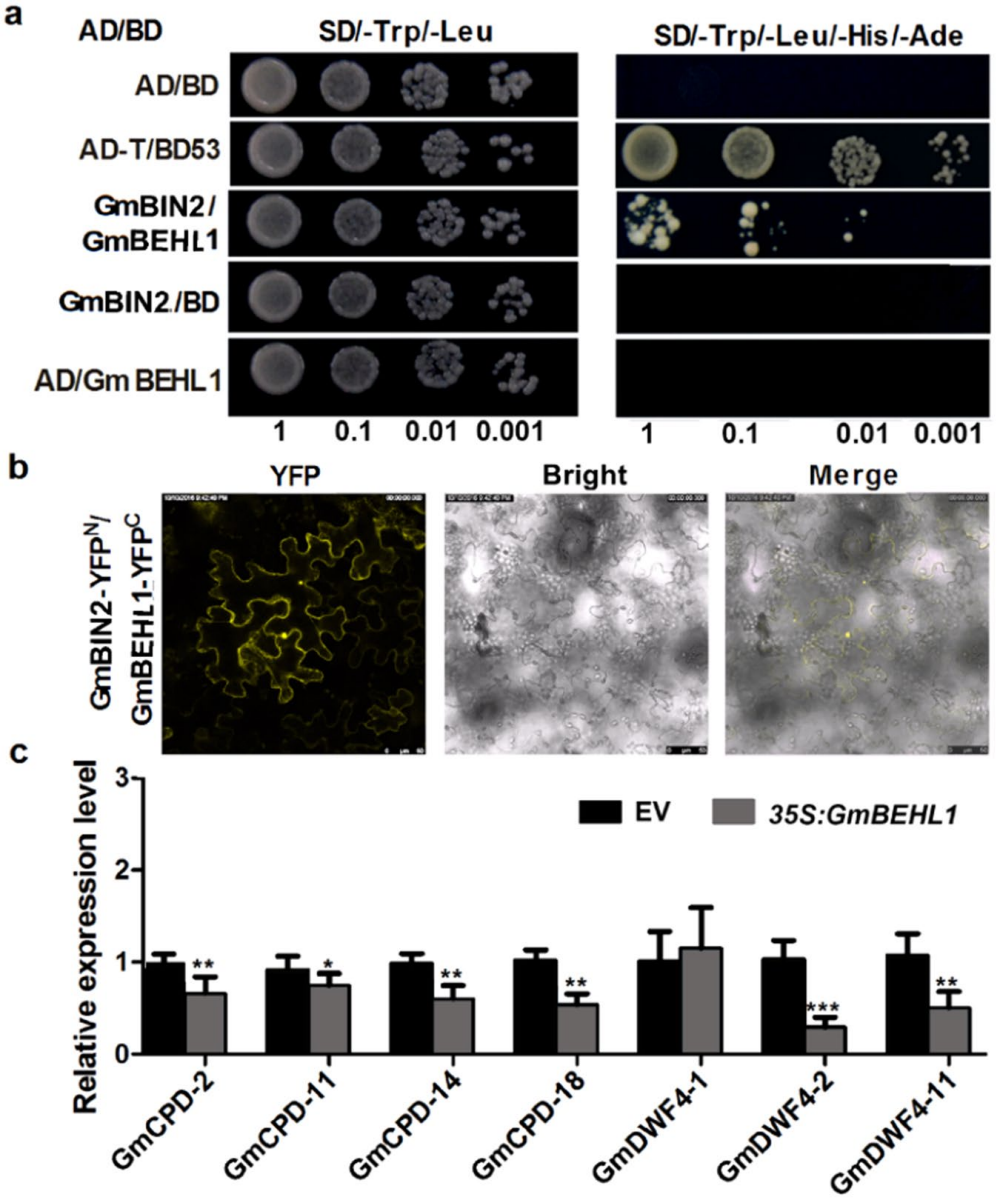
*GmBEHL1* encodes a functional ortholog of *Arabidopsis* BEH1. (**a** and **b**) GmBEHL1 interacts directly with GmBIN2. (**a**) Results of a Y2H assay to detect GmBEHL1 and GmBIN2L interactions. Yeast cells co-transformed with pGADT7/pGBKT7-GmBEHL1, pGADT7-GmBIN2/pGBKT7-GmBEHL1, and pGADT7-GmBIN2/pGBKT7 were grown on selective media lacking Leu and Trp (SD/−2) to check for transformation. The cells were subsequently grown on selective media lacking Ade, His, Leu, and Trp (SD/−4) to detect protein-protein interactions. (**b**) Results of a BiFC assay to detect the interaction of GmBEHL1 with GmBIN2. GmBIN2 and GmBEHL1 were fused to the N-terminus of YFP (nYFP) and C-terminus of YFP (cYFP), respectively. Bars = 25 μm. (**c**) qRT-PCR analysis of putative BR biosynthetic genes (*GmCPDs* and *GmDWF4s*) in roots transformed with EV1 and *35S:GmBEHL1* at 2 DAI (n = 6). The transcript amounts in each sample were normalized to those of *ELF1b*. The expression levels shown are the means ± SDs from three replicates. Asterisks represent statistically significant differences compared to empty vector (Student’s *t*-test; *p < 0.05, **p < 0.01, and ***p < 0.001).

In *Arabidopsis*, the protein stability and subcellular localization of BZR1 are regulated by a BIN2-mediated interaction and phosphorylation^43,44^. To test whether GmBEHL1 exhibits these same features, we cloned the *GmBIN2* (Glyma.13g228100) gene, which showed the highest level of protein sequence identity to *Arabidopsis* BIN2 (Fig. S4), and analyzed the interaction between GmBEHL1 and GmBIN2 by Y2H and BiFC approaches. As shown in Fig. 5a and b, GmBEHL1 exhibited a strong interaction with GmBIN2 in yeast cells and transformed *N. benthamiana* leaf cells, confirming that the subcellular localization of GmBEHL1 is regulated by the same mechanism as in *Arabidopsis*.

Previous work showed that BZR1/BEH1 is a transcriptional repressor that contains a DNA-binding domain and which binds directly to the promoters of feedback-regulated BR biosynthetic genes^36^. To test whether GmBEHL1 has the same effect on BR biosynthetic genes in soybean, we assessed the expression patterns of the putative soybean orthologs of *Arabidopsis Constitutive Photomorphogenesis and Dwarf* (*CPD*) and *Dwarf 4* (*DWF4*) (referred to as *GmCPDs* and *GmDWF4s*, respectively; Fig. S5) in *GmBEHL1OX* transgenic hairy roots. Our qRT-PCR results show that *GmBEHL1* overexpression caused a significant reduction in the expression of most of the *GmCPDs* and *GmDWF4s* tested compared with vector control roots (Fig. 5c). Thus, GmBEHL1 may also repress BR biosynthetic genes via a negative transcriptional feedback loop.

Previous studies have shown that the N-terminal sequence of BEH1 can bind to a BRRE (5′-CGTG[T/C]G-3′) in the promoters of its downstream target genes to regulate their expression^36^. To test whether GmBEHL1 has DNA-binding activity through the same domain, we made a construct to express the N-terminal sequence of GmBEHL1. The purified, truncated version of GmBEHL1 was used in an electrophoretic mobility shift assay (EMSA) to analyze the binding activity of the peptide to a BRRE-containing probe. As shown in Fig. S6, the N-terminus of GmBEHL1 bound directly to the probe, confirming that (like BES1 and its homologs) GmBEHL1 was able to bind the *cis*-regulatory element through its N-terminal domain. Together, these data suggest that GmBEHL1 is a functional ortholog of BEH1.

### Exogenous BRs reduced the nodule number but increased the nodule size in soybean

To assess the effects of BRs on soybean nodulation, we first examined the BR sensitivity of wild-type plants to 2, 4-epibrassinolide (eBL). Five-day-old plants were soaked in BD medium containing different concentrations of eBL (0, 0.01, 0.1, 0.5, and 1 μM) for 4 days, and then the hypocotyl lengths of the seedlings were measured. The hypocotyls of the young seedlings were sensitive to eBL; hypocotyl growth was significantly increased at 0.5 μM eBL (Fig. S7a and b). Since the epicotyls of the young seedlings were less sensitive to eBL, 9-day-old plants were treated with various concentrations of eBL. Elongated epicotyls were observed at eBL concentrations exceeding 0.1 μM (Fig. S7c and d). Since both the epicotyls and hypocotyls of young soybean seedlings showed the strongest response to eBL at a concentration of 0.5 μM (Fig. S7a–d) regardless of seedling age, 0.5 μM eBL was used in our subsequent rhizobial inoculation experiments.

**Figure 6:**
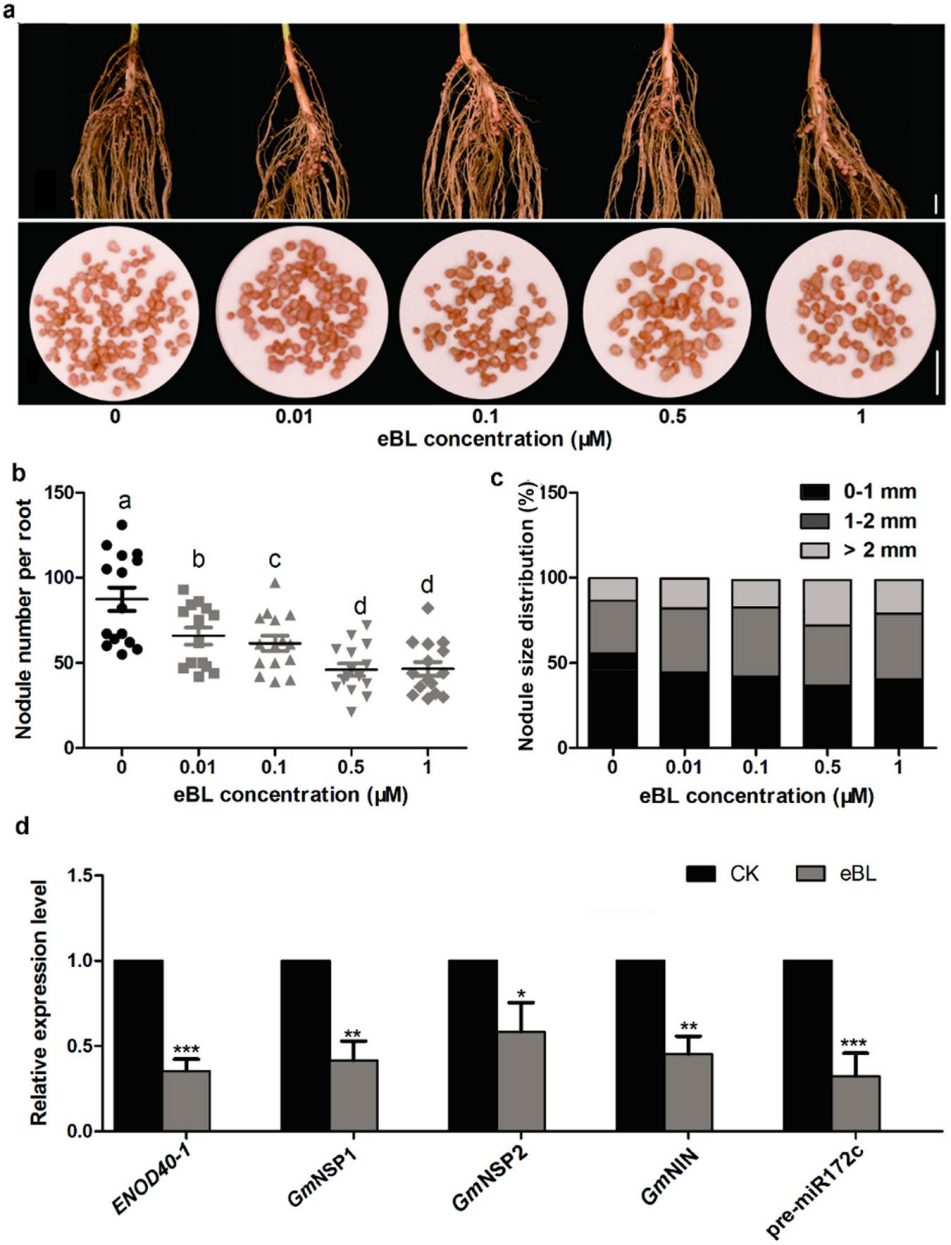
Effects of exogenous eBL on nodulation and the expression of nodulation-related genes. (**a**–**c**) Effects of exogenous eBL on soybean nodulation. Nine-day-old soybean seedlings were simultaneously treated with the indicated doses of eBL and *B. japonicum* USDA110 for 4 days; nodule number and size were then evaluated at 14 DAI. (**a**) The root system and nodulation phenotypes of the soybean seedlings (bars = 5 mm). (**b**) The average nodule number per treated plant. (**c**) A quantitative analysis of nodules of different sizes. (**d**) Results of the qRT-PCR analysis of *ENOD40-1*, *GmNSP1*, *GmNSP2*, *GmNIN*, and *pre-miR172c* expression in roots treated with 0.5 μM eBL (n = 12). The transcript amounts in each sample were normalized to those of *GmELF1b*. The expression levels shown are the means ± SDs from three replicates. Asterisks represent statistically significant differences compared to the control (Student’s *t*-test; *p < 0.05, **p < 0.01, and ***p < 0.001).

Next, we simultaneously treated 9-day-old soybean plants with various concentrations of eBL and *B. japonicum* strain USDA110 for 3 days and then transplanted them to pots for further growth and nodulation. At 2 weeks after eBL treatment, a substantially reduced number of nodules was observed compared with eBL-untreated plants (Fig. 6a and b). Interestingly, the eBL-treated plants exhibited fewer and larger nodules as the eBL concentration increased, suggesting that BRs have inhibitory and promoting effects on nodule initiation and development, respectively (Fig. 6c). To explore the regulatory roles of BRs in soybean nodulation further, we analyzed the expression levels of several marker genes belonging to the NF signaling pathway in eBL-treated plants. As shown in Fig. 6d, the rhizobia-induced upregulation of *ENOD40-1*, *GmNSP1*, *GmNSP2*, *GmNINa*, and *pre-miR172c* (NF signaling pathway) was markedly repressed in plants treated with eBL compared with control plants. These results suggest that BRs interact antagonistically with the NF signaling pathway to regulate nodule formation in soybean.

## Discussion

Nodulation is a complex process involving two tightly coupled steps: rhizobial infection and nodule organogenesis. Successful establishment of a symbiosis between rhizobia and plants is precisely controlled by endogenous cues and environmental conditions. Although the molecules and mechanisms that participate in these processes are unclear, there is increasing evidence that phytohormones integrate relatively independent but closely related biological processes during legume nodulation. To date, it has been shown that most phytohormones mediate nodulation. It is conceivable that multiple phytohormones modulate nodule development antagonistically or synergistically in legumes. In recent decades, it has been reported that auxin, CK, ABA, and ethylene play crucial roles in legume nodulation, but it is unknown how the rest of the phytohormones (e.g., BRs) act during nodulation and how they interact to ensure successful nodulation. In this study, we found that BRs exert opposite effects on nodule number and size in soybean, and we identified *GmBEHL1* as an NNC1-interacting protein that regulates soybean nodulation.

BES1/BRZ1 family proteins are central transcription factors in the BR signaling pathway that regulate plant responses to BRs by targeting many genes related to plant growth and stress tolerance^41,45^. It has been shown that BZR1 and BES1 (BZR2) function redundantly with their homologs BEH1-4 in the *Arabidopsis* BR signaling pathway^42^. Recently, an analysis of an *Arabidopsis* BZR1-like gene in soybean (*GmBZL2*) revealed that GmBZLs are highly conserved with *Arabidopsis* BZR1 in the BR signaling pathway^46^. Here, we provide further evidence that *GmBEHL1* is an ortholog of *Arabidopsis BEH1*. GmBEHL1 shares a high level of amino acid sequence identity with *Arabidopsis* BEH1 and contains the typical BES1_domain of BES1/BZR1 family proteins. Further, GmBEHL1 is localized to both the nucleus and cytoplasm, implying that it can shuttle from the cytoplasm to the nuclues in response to BRs. Importantly, GmBEHL1 can bind directly to BRRE-containing DNA fragments and it is able to interact with GmBIN2, a homolog of *Arabidopsis* BIN2, a GSK3-like kinase. These data suggest that GmBEHL1 is a key regulator of the BR signaling pathway in soybean. Determining whether BRs induce GmBEHL1 dephosphorylation, and whether the dephosphorylated protein is localized to the nucleus, will provide convincing evidence for the role of GmBEHL1 in mediating BR signaling.

Importantly, our results show that GmBEHL1 mediates soybean nodulation. Firstly, GmBEHL1 interacts with NNC1. We previously demonstrated that NNC1 is the target of miR172c and that it negatively modulates soybean nodulation by directly repressing *ENOD40* transcription^22^. The GmBEHL1-NNC1 interaction implies that GmBEHL1 participates in the regulation of nodulation as a co-repressor of NNC1. Secondly, *GmBEHL1* exhibits a unique expression pattern during nodule primordia formation, nodule development, and nodule maturation, indicating multiple roles for the gene in symbiotic nodulation. Thirdly, our genetic data support the notion that *GmBEHL1* negatively regulates nodulation through the classical NF signaling pathway because the alteration of *GmBEHL1* affected the expression of several marker genes in the nodulation pathway. Finally, *GmBEHL1* may affect the functionality of nitrogen-fixing nodules because *GmBEHL1* was expressed in the nitrogen fixation zone of functioning nodules.

In *Arabidopsis*, BZR1 interacts with other transcription factors to co-repress target gene expression^47^. Since both NNC1 and GmBEHL1 are negative regulators of nodulation, it is possible that NNC1 and GmBEHL1 regulate soybean nodulation by acting as co-repressors of target genes. NNC1 is an AP2 transcription factor family member that represses *ENOD40* transcription^22^. However, the 2-kb promoters of *ENOD40* genes do not contain typical *cis*-regulatory BRREs (data not shown). Because both AP2 and BES1/BZR1 family transcription factors have many target genes in plant genomes, NNC1 and GmBEHL1 likely co-repress the transcription of other genes to mediate nodule organogenesis and nitrogen fixation in mature nodules. The fact that *GmBEHL1* expression affects many marker genes, including *GmNSPs* and *GmNINa*, suggests that GmBEHL1 exerts its regulatory effect at multiple levels of soybean nodulation, beginning at the early stages of infection and nodule organogenesis. Thus, we also do not exclude the possibility that GmBEHL1 complexes with other transcriptional regulators to excert it functions during nodulation. The application of ChIP-seq technology will facilitate the identification of genes targeted by NNC1 and GmBEHL1 and help elucidate the novel molecular mechanism underlying the NNC1/GmBEHL1-mediated regulation of nodulation in soybean. Since BES1/BZR1 family proteins have redundant roles in various biological processes, it is conceivable that these proteins dynamically and coordinately regulate several processes involved in soybean nodulation.

Based on our data, it appears that NNC1 and GmBEHL1 mediate crosstalk between the nodulation and BR signaling pathways to orchestrate nodule organogenesis in soybean roots. BRs are important regulators of plant growth and development^33^; however, the roles of BRs in soybean nodulation remain unclear. Previous studies showed that BR treatment did not affect nodulation in the soybean cultivar Enrei^40^. In this study, we treated the soybean reference cultivar Williams 82 with various concentrations of eBL. Unexpectedly, exogenous treatment with eBL reduced the nodule number but enlarged the nodules in the plants. Our data show that BRs lower the nodule number in soybean by antagonistically regulating the NF signaling pathway (BR treatment repressed the expression of all positive regulators of the NF signaling pathway). However, we still do not know how BRs cause nodule enlargement. Given the role of BRs in cell division and proliferation, it is possible that BR treatment enhances cell cycle progression. Elucidation of the mechanism underlying the control of nodule size by BRs will further our understanding of how nodule organ size is maintained in legumes. We speculate that the different responses of cultivars Williams 82 and Enrei to BRs are largely due to the BR treatment protocols used; however, we cannot exclude the possible impact of their genetic backgrounds.

In summary, our data demonstrate that the BR signaling pathway plays diverse roles in soybean nodule organogenesis and nodule size regulation. We identified GmBEHL1 as a potential factor that mediates crosstalk between the BR and NF signaling pathways, possibly through an interaction with NNC1. To the best of our knowledge, this is the first study to establish a direct link between the nodulation pathway and BR signaling pathway in soybean. Our findings provide novel insight into the regulation of soybean nodulation by BRs and will facilitate the molecular breeding of new soybean varieties with improved symbiotic nitrogen fixation efficiency.

## Methods

### Plant and rhizobia growth, hairy root transformation, and *B. japonicum* inoculation

Soybean [*G. max* (L.) Merrill cv. Williams 82] was used to clone and analyze *GmBEHL1*. Soybean seedlings were cultured under 16 h/8 h light/dark conditions in a growth room at 25–26 °C and inoculated with *B. japonicum* strain USDA110 for nodulation pheonotype analyses as described previously^22^. Briefly, each young soybean seedling was inoculated with 30 ml of bacteria suspended in distilled water. For RNA extraction, plant materials were rinsed briefly in phosphate-buffered saline (pH 7.5) to remove vermiculite. All harvested marterials were then immediately frozen in liquid nitrogen and stored at −80 °C for RNA extraction. For soybean hairy root transformation, healthy and uniform soybean seeds were sterilized with chlorine gas for 12–14 h. The sterilized seeds were then germinated in B5 medium for 4 days under 16 h/8 h light/dark conditions in a growth chamber at 25–26 °C. Germinating seedlings were used for hairy root transformation as described previously with *Agrobacterium rhizogenes* strain K599^48^. For all nodulation assays, transgenic composite plants or soybean seedlings were transplanted to pots (8 × 8 cm) containing vermiculite and irrigated with a nitrogen-deficient solution as described elsewhere^22^. The plants were grown for 1 week (16 h of light at 25 °C and 50% relative humidity) to allow rooting and acclimation to the enviroment. The plants were then inoculated with a suspension of *B. japonicum* strain USDA110 (OD_600_ = 0.08). Nodule phenotypes, including nodule number and nodule size, were evaluated at 28 DAI.

### Y2H assay

The full-length coding sequences of *GmBEHL1* and *NNC1* were amplified using the listed primers (Supplemental Table 1). Gateway PCR products were cloned into the vectors pGBKT7 and pGADT7 by BP and LR reactions. Y2H assays were done according to the Matchmaker GAL4 Two-Hybrid System (Clontech, Mountain View, CA). Yeast transformants were exhaustively selected on SD/-Ade/-His/-Leu/-Trp (SD/−4) medium. The constructs used to validate protein-protein interactions were cotransformed into *Saccharomyces cerevisiae* strain AH109. Suspended, transformed yeast (5 μl) were spread onto plates containing SD/-Ade/-His/-Leu/-Trp medium, and protein-protein interactions were judged based on the growth of the yeast after 2–3 days of incubation at 30 °C.

### BiFC assays

The coding sequences of *GmBEHL1* and *NNC1* were cloned into the N-terminus and C-terminus of YFP through the Gateway reaction using the pDONOR vector system (Invitrogen, Carlsbad, CA), respectively. The primers used are listed in Supplemental Table 1. The resulting constructs were transformed into *Agrobacterium* strain GV3101 for transient expression of the proteins in *N. benthamiana* leaf cells^22^. The *N. benthamiana* plants were cultured for at least 36 h; YFP fluorescence was observed using a Leica Microsystems confocal laser scanning microscope (Wetzlar, Germany).

### DNA extraction from and PCR-based analysis of the transgenic roots

DNA from the hairy roots of transgenic composite plants or soybean seedlings was extracted as described previously^49^ and used for detection of the *Bar* gene by PCR using the primers listed in Supplemental Table 1.

### RNA extraction and quantitative PCR analysis

Total RNAs were extracted from transgenic hairy roots or soybean seedlings using TRIpure Reagent (Aidlab Biotechnologies Ltd., Beijing, China). The RNA samples were then treated with gDNA Wiper Mix (Vazyme Biotech Co., Ltd., Nanjing, China) to remove contaminating genomic DNA. cDNA strands were synthesized from the RNAs using a FastQuant RT Kit (Vazyme Biotech Co., Ltd.). qRT-PCR was done using SuperReal PreMix Plus (Vazyme Biotech Co., Ltd.) with gene-specific primers (Table S1). *GmELF1b* was used as an internal control.

### Vector construction

For the *GmBEHL1 promoter:GUS* reporter construct, the putative promoter region (2,000 bp) of *GmBEHL1* was amplified from cv. Williams 82 genomic DNA and cloned into a T-vector by the BP reaction for sequencing. Positive plasmids (T-vector containing the *GmBEHL1* promoter sequence) were used to generate the construct pCAMBIA1391-*GmBEHL1pro:GUS* through the LR reaction. For the *35S:GmBEHL1* construct and the constructs for the Y2H (BD-GmBEHL1) and BiFC (GmBEHL1-YFP^**N**^) assays, the coding DNA sequence of *GmBEHL1* was amplified and cloned into pDORNOR207 by the BP reaction for sequencing, and positive plasmids (pDORNOR207 with the *GmBEHL1* coding DNA sequence) were used to generate the constructs by the LR reaction. The primers used for plasmid construction are listed in Table S1.

### Histochemical analysis of *GmBEHL1* transcription

Composite transgenic plants expressing *GmBEHL1pro:GUS* were generated through *A. rhizogenes*-mediated hairy root transformation. The transformed hairy roots of the composite seedlings were stained with X-Gluc to test for β-glucuronidase activity before and after inoculation with *B. japonicum* strain USDA110 at the specified time points.

### EMSAs

EMSAs were performed as described previously^22^ using a Light Shift Chemiluminescent EMSA Kit (Pierce, Rockford, IL) according to the manufacturer’s protocol. Briefly, MBP-tagged nGmBEHL1 (amino acids 10–91) was expressed in *Escherichia coli* BL21 cells. The probe-binding activity of the protein was analyzed using oligonucleotides labeled with biotin at the 5′ end (Invitrogen). As competition, 200-fold unlabeled probe was added to the reactions.

### Brassinolide treatment

To examine the response of soybean to BR treatment, 5-day-old seedlings germinated and grown in vermiculite were harvested for root treatment with BD media containing different concentrations (0, 0.01, 0.1, 0.5, and 1 μM) of eBL (Realtimes Beijing Technology Co., Ltd., Beijing, China). The lengths of the hypocotyls were measured at 4 days after treatment. To confirm the BR response of Williams 82, 9-day-old seedlings were treated with eBL and the epicotyl lengths were measured as described above. For the nodulation assay, 9-day-old seedlings treated simultaneously with eBL and a rhizobia inoculum for 4 days were transferred to vermiculite for further growth, and the number of nodules was evaluated 2 weeks after transplanting. For the expression analysis of marker genes in the NF signaling pathway, 5-day-old seedlings germinated in 50-ml centrifuge tubes containing vermiculite were watered with 0.5 μM eBL and the roots were collected for RNA extraction and qRT-PCR analysis.

### Statistical analysis

Data analysis was done using GraphPad Prism 5 (GraphPad Software, Inc., La Jolla, CA). The means and standard deviations (SDs) of the data were calculated. Student’s *t*-test and an analysis of variance were applied to generate *p*-values. Student-Newman-Kuels tests were conducted when statistically significant differences existed.

## Electronic supplementary material

Supplementary Information
