# Supplementary material for: GmBEHL1, a BES1/BZR1 family protein, negatively regulates soybean nodulation
Source: Sci Rep. 2018 May 16;8:7614. doi: 10.1038/s41598-018-25910-x (PMC5955893; doi:10.1038/s41598-018-25910-x)
Supplement: Supplementary file 1 — Supplementary Information [file 41598_2018_25910_MOESM1_ESM.docx]

**GmBEHL1, a BES1/BZR1 family protein, negatively regulates soybean nodulation**

Qiqi Yan^1,§^, Lixiang Wang^1,§^, and Xia Li^1,*^

^1^State Key Laboratory of Agricultural Microbiology, College of Plant Science and Technology, Huazhong Agricultural University, Wuhan 430070, P.R. China

^§^These authors contributed equally to this work.

**Corresponding author**

Xia Li

E-mail: xli@mail.hzau.edu.cn

Telephone number: 86-027-87856638

**Supplementary information**


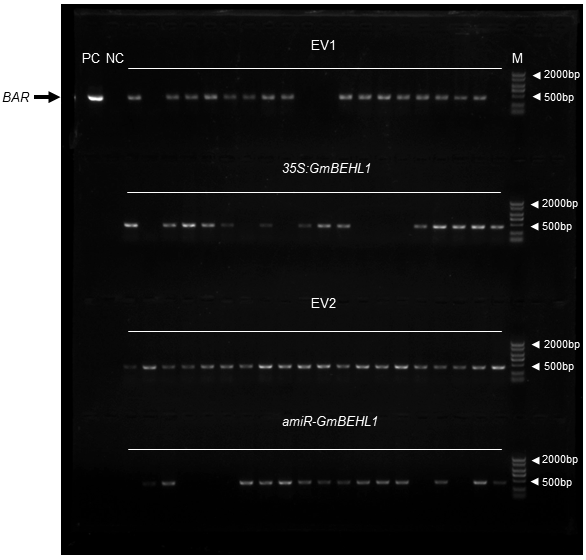


**Fig. S1** Transgenic validation of the hairy roots of the composite soybean plants. The putative hairy roots expressing EV1 (empty vector 1) , *35S:GmBEHL1* and EV2, *amiR-GmBEHL1* at 28 DAI were collected for DNA extraction and then the *BAR* were detected by PCR, and the positive hairy roots were selected for RNA extraction and gene expression analysis. PC: positive control using the EV DNA containing the *bar* gene; NC: negative control using DNA from the untransformed roots; M: DL2000 DNA ladder.


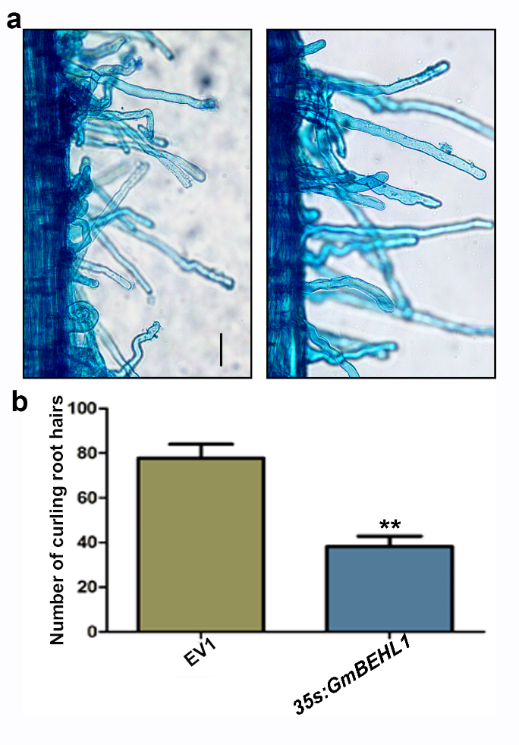


**Fig. S2** Overexpression of *GmBEHL1* decreases the number of deformed root hairs.

At 6 DAI, 2 cm root segments of hairy roots overexpressing *GmBEHL1* or expressing EV1 below the root-hypocotyl junction were cut and stained with 1% (w/v) methylene blue. Deformed root hairs were counted (n = 10 to 12). (a) Root hair deformation in transgenic roots harboring EV1 and *35S:GmBEHL1* vector. Bar = 40 μm. (b) Quantification of deformed root hairs in the transgenic lines (n=10 to 12). Values are averages ± SD from three independent experiments. Asterisks represent statistically significant differences, (Student’s *t* test, ** *p*< 0.01).


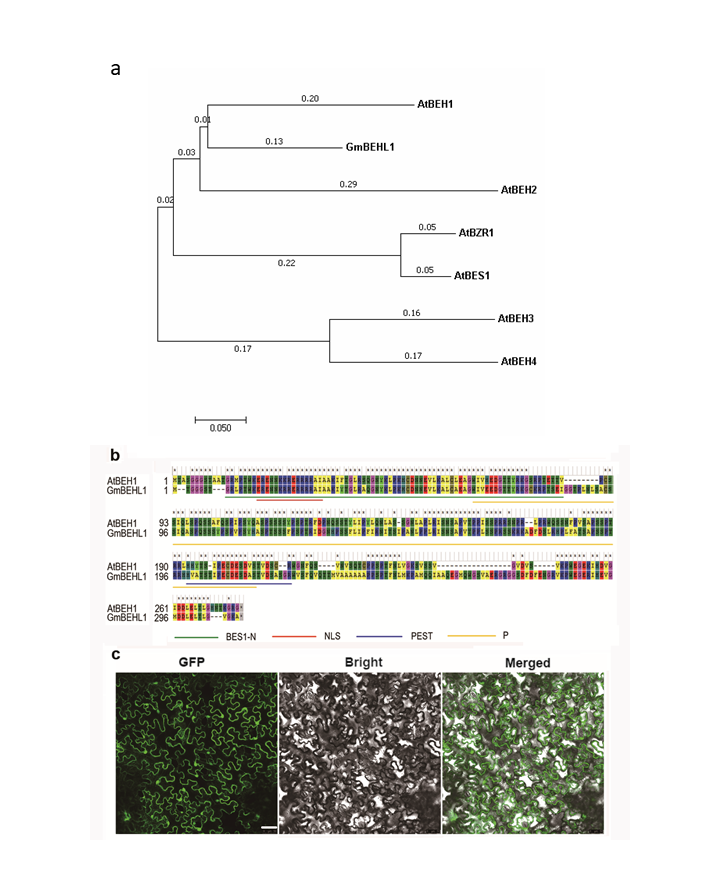


**Fig. S3** Protein structural characteristics of GmBEHL1. (a) Phylogenetic analysis of GmBEHL1 and Arabidopsis homologs; (b) Protein alignments and domain analysis of GmBEHL1 and Arabidopsis BEH1. The protein sequences used in phylogenetic and domain analysis were download from NCBI (<https://www.ncbi.nlm.nih.gov/>) and Phytozome. (c) Subcellular localization of the GmBEH1L protein. The GmBEHL1-GFP fusion protein was expressed in N. *benthamiana* leaf cells and the GFP fluorescence was visualized in confocal laser microscope. Bar = 50 μm.


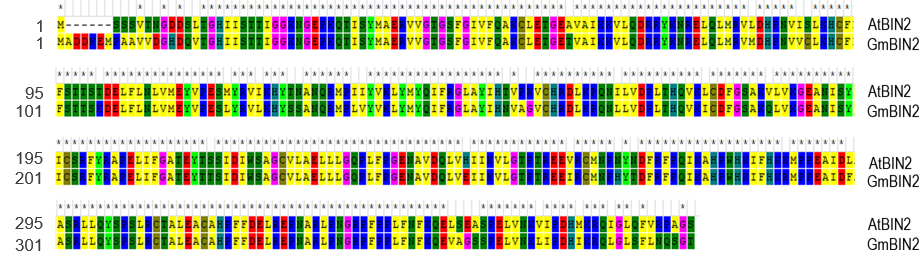


**Fig. S4** Amino acid sequence alignment of Arabidopsis BIN2 and GmBIN2. The homolog blast was conducted to serach GmBIN2, the Protein sequences of Arabidopsis BIN2 and GmBIN2 (Glyma.13g228100) were downloaded from NCBI (<https://www.ncbi.nlm.nih.gov/>), and the MEGA 5 was applied for the sequence alignment. GmBIN2 shares 88% sequence identity.


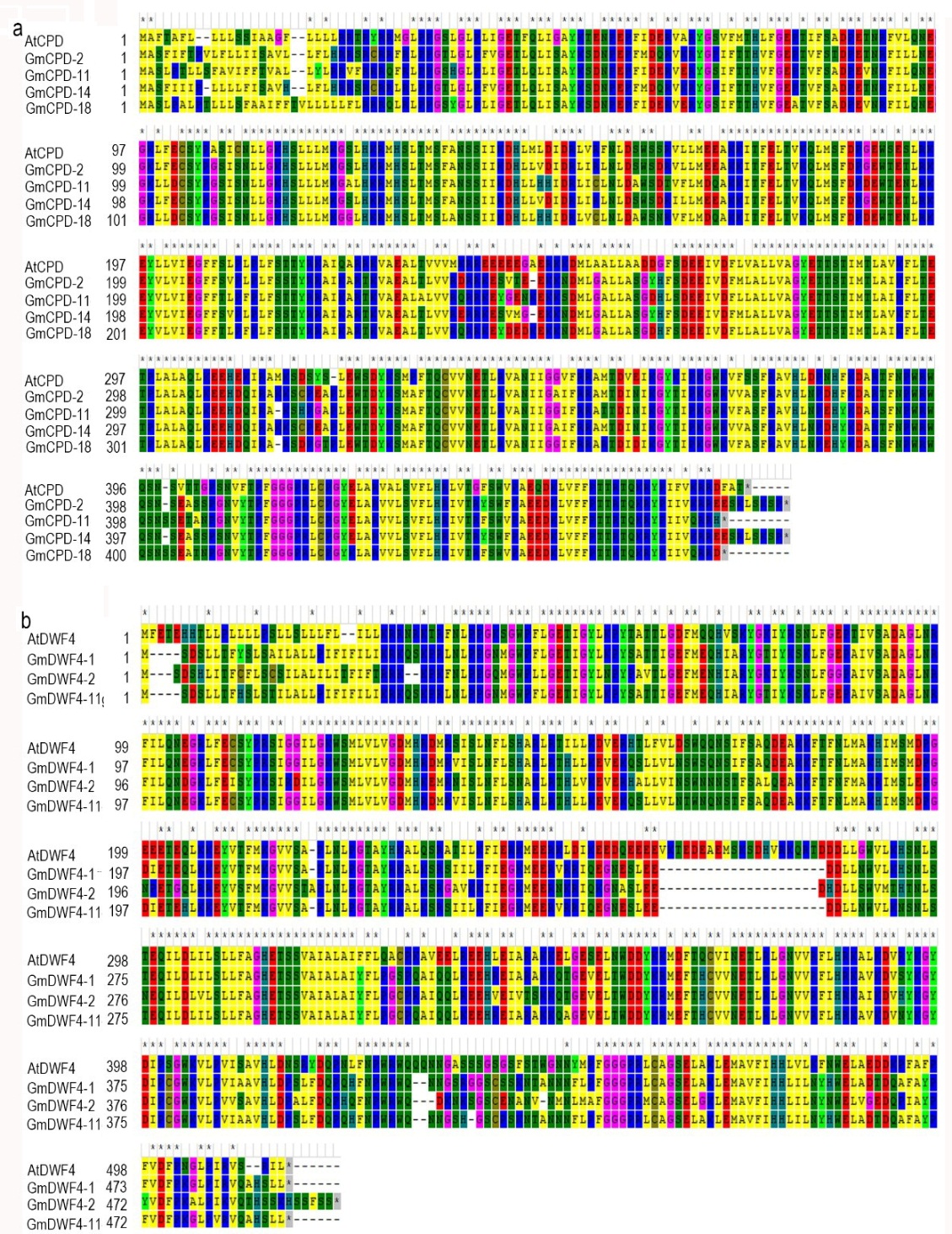


**Fig. S5** Amino acid sequence alignment of soybean homologs of Arabidopsis CPD and DWF4. CPD (AT5G05690) and DWF4 (AT3G50660) protein sequences of Arabidopsis and their putative soybean orthologs GmCPDs (Glyma.02G256800, Glyma.11G228900, Glyma.14G059900 and Glyma.18G028300), GmDWF4s (Glyma.01G175500, Glyma.02G057500 andGlyma.11G067700) were downloaded from Phytozome (https://phytozome.jgi.doe.gov/pz/portal.html) and TAIR ([www.arabidopsis.org/](http://www.arabidopsis.org/) ), the MEGA5 was applied for the sequence alignment.


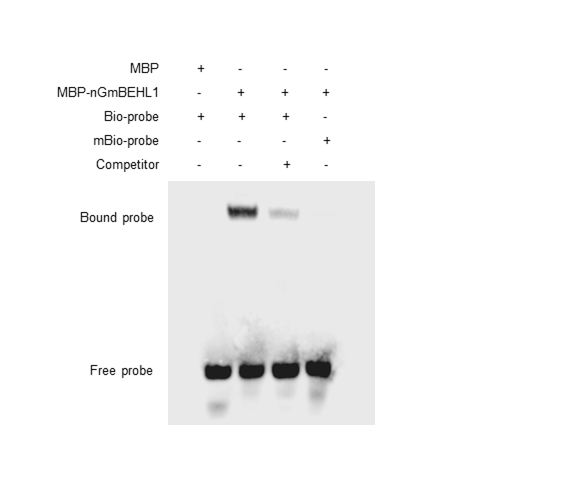


**Fig. S6** DNA binding activity of GmBEHL1 shown by ENMS result. Biotin-labelled DNA probe containing BBRE motif(Bio-probe) were incubate with MBP-nGmBEHL1 (N-termini of GmBEHL1(10-91aa) which contains the BES1 protein domain) fusion protein, and the free and bound probes were separated on an acrylamide gel (lane 2). Lane 3 shows the competition for binding using 200× competitive probes. MBP was used as a negative control (lane 1). mBio-probe (mutated Bio-probe), biotin-labelled probe containing mutated nucleotides from 5′-CACGTG-3′ to 5′-AAAAAA-3′, was used as another negative control(lane 4). Three biological replications were performed.


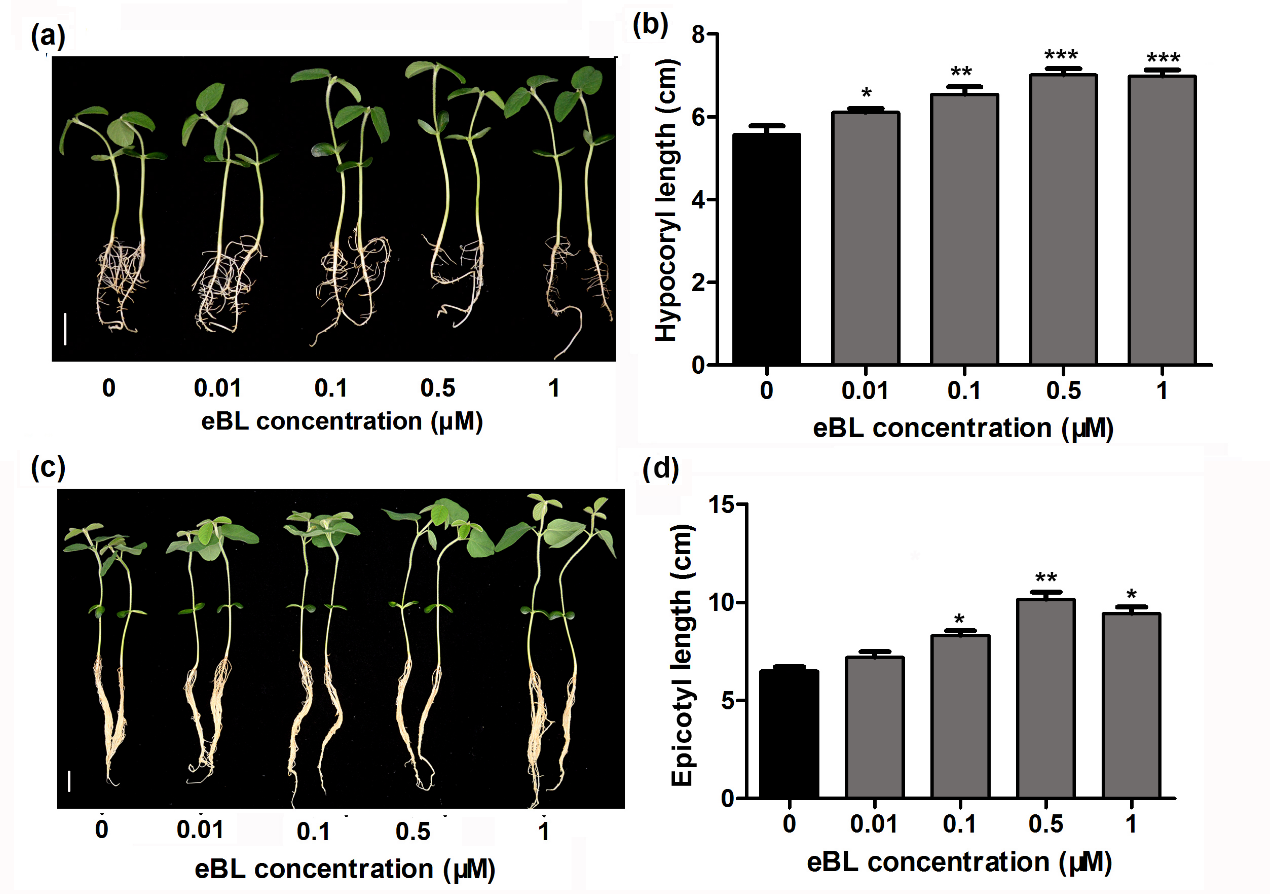


**Fig. S7** Effects of exogenous eBL on the soybean growth. (a) and (b) Five days-old seedlings were soaked in low nitrogen liquid solution supplemented with 0, 0.01, 0.1, 0.5 and1.0 μM eBL for 4 days, the pictures of representative seedlings were taken (a) and the hypocotyl length of soybean seedlings were measured (b). (c) and (d) nine day-old young seedlings were soaked in low nitrogen liquid solution supplemented with 0, 0.01, 0.1, 0.5 and 1.0 μM eBL for 4 days, and the pictures of representative plants were shown (c), and the epicotyl length of the seedlings were measured. Error bars represent the standard error of the means (n =12). Means with different letters are significantly different (*p*< 0.05; Tukey’s test). bars = 2 cm.

**Table S1**

Primers used in the study

The primers for Real-time PCR

| Primer | Primer sequences |
| --- | --- |
| *qRT-ELF1b* | Forward: GTTGAAAAGCCAGGGGACA |
| *qRT-ELF1b* | Reverse: TCTTACCCCTTGAGCGTGG |
| *qRT-ENOD40-1* | Forward: TCTCTCTTGAGTGGCAGAAGCA |
| *qRT-ENOD40-1* | Reverse: TGGAGTCCATTGCCTTTTCG |
| *qRT-NIN* | Forward: TGGCGCACCATGCTAACAT |
| *qRT-NIN* | Reverse: GGGTGTCATGGCAATCCTTT |
| *qRT-NSP1* | Forward: GGTCTATAACTTTTGCTTCCAGC |
| *qRT-NSP1* | Reverse: CAGTGTCTTCGCCAAGAACTTG |
| *qRT-NSP2* | Forward: ACGACGCCGACGAAGAAA |
| *qRT-NSP2* | Reverse: AGACCAACTCCTTGAGCCGA |
| *qRT-RIC1* | Forward: CAAATGCAACAATGGCTACTCG |
| *qRT-RIC1* | Reverse: GCCATGGAGATTACTAGCCTGC |
| *qRT-RIC2* | Forward: GGCCACAATCCATTATTCGCT |
| *qRT-RIC2* | Reverse: ACGCACACGCTTTGATAGGTG |
| *qRT-pre-miR172c* | Forward: ATCAGTCACTGTTTGCCGGT |
| *qRT-pre-miR172c* | Reverse: GTCATTTATTGCTGCTGCAG |
| *qRT-CPD-2* | Forward: GAAAGCGTAACGGAGGAGAA |
| *qRT-CPD-2* | Reverse: GACGAGCAAAGCCAACATAAA |
| *qRT-CPD-11* | Forward: CACCTTTCCGACGAGGAAATA |
| *qRT-CPD-11* | Reverse: TGAGGAACTTGATCGCAAGAG |
| *qRT-CPD-14* | Forward: GTTGCTTCATTTCGTGCGGT |
| *qRT-CPD-14* | Reverse: GTGGACCTCCTCCAAAAGGG |
| *qRT-CPD-18* | Forward: GAAGCAACAAACCCTGGTAATG |
| *qRT-CPD-18* | Reverse: GGTGAAGGAAGACGGAAAGT |
| *qRT-DWF4-1* | Forward: GGCATTGAAGTCTCGGTCCA |
| *qRT-DWF4-1* | Reverse: GACGAAGTTTCATGGCCAGC |
| *qRT-DWF4-2* | Forward: GGCATGCCCTTCTCGTTATTA |
| *qRT-DWF4-2* | Reverse: CCAGGCTCCAAGCTCATTATAC |
| *qRT-DWF4-11* | Forward: GTCTGACTCACTCCTAACTTTCC |
| *qRT-DWF4-11* | Reverse: CCTGGGTTTGCTTTGCTTTC |
| *qRT-BEHL1* | Forward: TGGAGGAACACCACTGAAC |
| *qRT-BEHL1* | Reverse: TGGGATGAGAAAGGAAGAAG |
|  |  |

The primers used for vector construction

| Vector | Primer sequences |
| --- | --- |
| *35S:BEHL1* | Forward-gateway: GGGGACAAGTTTGTACAAAAAAGCAGGCTTC ATGACCGGCGGCGGATC |
|  | Reverse-gateway: GGGGACCACTTTGTACAAGAAAGCTGGGTC AGCCTTTCCAACTCCTAGAG |
| *amiR-BEHL1* | s: GATATCTCACTCGTGGGTCGCTTTCTCTCTTTTGTATTCC |
|  | a: GAAAGCGACCCACGAGTGAGATATCAAAGAGAATCAATGA |
|  | *s: GAAAACGACCCACGACTGAGATTTCACAGGTCGTGATATG |
|  | *a: GAAATCTCAGTCGTGGGTCGTTTTCTACATATATATTCCT |
|  | pA: CTGCAAGGCGATTAAGTTGGGTAAC |
|  | pB: GCGGATAACAATTTCACACAGGAAACAG |
|  | pAgateway:GGGGACAAGTTTGTACAAAAAAGCAGGCTTCCTGCAAGGCGATTAAGTTGGGTAAC |
|  | pBgateway:GGGGACCACTTTGTACAAGAAAGCTGGGTCGCGGATAACAATTTCACACAGGAAACAG |
| BEHL1-N-MBP | Forward-EcoRⅠ: CGGAATTCAGGTTGCCGACGTGGAAG |
|  | Reverse-SalⅠ: ACGCGTCGACTAAGTTCAGTGGTGTTCCTC |
| *proBEHL1:GUS* | Forward-HindⅢ: CCCAAGCTTCGCCGCAGAAATGGACTGT |
|  | Reverse-BamHⅠ: CGGGATCCCGTTCCGGCGTGACGAAAC |
| *35S:BIN2* | Forward-gateway: ATGTCTTCCTCTGTCACCAAT |
|  | Reverse-gateway: TGATCCTGCCGGACGAACA |

Probes used for EMSA experiment

| Probe | Probe sequences |
| --- | --- |
| *wt-Probe* | \| Forward: AAGGGTATTACATATCACACGTGGAAATCTTCCACCTT \| \| --- \| |
| *wt-Probe* | Reverse : AAGGTGGAAGATTTCCACGTGTGATATGTAATACCCTT |
| *m-Probe* | Forward: AAGGGTATTACATATAAAAAATGGAAATCTTCCACCTT |
| *m-Probe* | Reverse: AAGGTGGAAGATTTCCATTTTTTATATGTAATACCCTT |

Other primers

| Primer | Primer sequences |
| --- | --- |
| *Bar* | Forward: CTACATCGAGACAAGCACGGTCAA |
| *Bar* | Reverse: AGAAACCCACGTCATGCCAGTTC |
